# Effects of Anabolic Steroids and High-Intensity Aerobic Exercise on Skeletal Muscle of Transgenic Mice

**DOI:** 10.1371/journal.pone.0080909

**Published:** 2013-11-15

**Authors:** Karina Fontana, Gerson E. R. Campos, Robert S. Staron, Maria Alice da Cruz-Höfling

**Affiliations:** 1 Department of Pharmacology, Faculty of Medical Sciences, University of Campinas, Campinas, Brazil; 2 Department of Histology and Embryology, Institute of Biology, University of Campinas, Campinas, Brazil; 3 Department of Biologia Estrutural e Funcional, Institute of Biology, University of Campinas, Campinas, Brazil; 4 Department of Biomedical Sciences, Heritage College of Osteopathic Medicine, Ohio University, Athens, Ohio, United States of America; University of Pittsburgh, United States of America

## Abstract

In an attempt to shorten recovery time and improve performance, strength and endurance athletes occasionally turn to the illicit use of anabolic-androgenic steroids (AAS). This study evaluated the effects of AAS treatment on the muscle mass and phenotypic characteristics of transgenic mice subjected to a high-intensity, aerobic training program (5d/wk for 6 weeks). The transgenic mice (CETP^+/-^LDLr^-/+^) were engineered to exhibit a lipid profile closer to humans. Animals were divided into groups of sedentary (Sed) and/or training (Ex) mice (each treated orally with AAS or gum arabic/vehicle: Sed-C, Sed-M, ex-C, ex-M). The effects of AAS (mesterolone: M) on specific phenotypic adaptations (muscle wet weight, cross-sectional area, and fiber type composition) in three hindlimb muscles (soleus:SOL, tibialis anterior:TA and gastrocnemius:GAS) were assessed. In order to detect subtle changes in fiber type profile, the entire range of fiber types (I, IC, IIAC, IIA, IIAD, IID, IIDB, IIB) was delineated using mATPase histochemistry. Body weight gain occurred throughout the study for all groups. However, the body weight gain was significantly minimized with exercise. This effect was blunted with mesterolone treatment. Both AAS treatment (Sed-M) and high-intensity, aerobic training (ex-C) increased the wet weights of all three muscles and induced differential hypertrophy of pure and hybrid fibers. Combination of AAS and training (ex-M) resulted in enhanced hypertrophy. In the SOL, mesterolone treatment (Sed-M and ex-M) caused dramatic increases in the percentages of fiber types IC, IIAC, IIAD, IID, with concomitant decrease in IIA, but had minimal impact on fiber type percentages in the predominantly fast muscles. Overall, the AAS-induced differential adaptive changes amounted to significant fiber type transformations in the fast-to-slow direction in SOL. AAS treatment had a significant effect on muscle weights and fiber type composition in SOL, TA and GAS which was even maximized in animals subjected to metabolically high-intensity aerobic exercise.

## Introduction

Anabolic-androgenic steroids (AAS) are synthetic derivatives of testosterone which have been chemically modified to maximize anabolic effects and minimize undesirable androgenic effects [1]. Given the powerful anabolic effect, AAS have been prescribed for patients with hypogonadism, as well as those debilitated by chronic diseases and/or severe muscle catabolism [2]. Although banned from sports, the illicit use of AAS by professional and recreational athletes continues despite a long list of serious side effects [3]. In general, these athletes are interested in decreasing body fat while increasing muscle mass and strength in an effort to enhance physical performance [4]. 

Skeletal muscle has the ability to respond to new endogenous and exogenous physiological demands by changing its phenotypic characteristics [5]. The large diversity of myosin heavy chain (MHC) isoforms expressed in muscle helps to form the basis for this remarkable plasticity. It is the expression and co-expression of these various MHC isoforms within a given fiber that ultimately delineates the entire range of fiber types [6-8]. The prevalence of certain types of these fibers accounts for the functional and structural characteristics of a given muscle, and hence its phenotype. 

The present study was undertaken to examine the effects of mesterolone (an anabolic-androgenic steroid) on the fiber type composition and cross-sectional area of skeletal muscle fibers of sedentary and high-intensity, aerobically-exercised transgenic mice. Thus, the aim was to investigate the role of mesterolone in a supposed catabolic environment. Does the interaction of anabolic hormone treatment and high-intensity aerobic exercise produce an increase in muscle mass and redistribution of skeletal muscle fiber types? Are muscles with distinct metabolic and contractile properties (i.e., fast/glycolytic vs. slow/oxidative) differently modulated by the anabolic-androgenic steroid treatment combined with a high-intensity, endurance-type training program? As such, three skeletal muscles were studied under these experimental conditions: soleus (SOL), tibialis anterior (TA) and gastrocnemius (GAS). The entire range of pure and hybrid fiber types were delineated using myofibrillar adenosine triphosphatase (mATPase) histochemical methods allowing the detection of subtle changes in type composition. The mice used in the current study were genetically engineered to exhibit a lipid profile closer to humans allowing for a more relevant comparison. 

## Materials and Methods

### Animals

The experimental protocol was approved by the University’s Committee for Ethics in Animal Use (CEUA/UNICAMP, Protocol 700-1)) and followed the "Principles of Laboratory Animal Care" (NIH publication no. 85-23, revised 1996). The transgenic mice used in this study have been described in detail elsewhere [9,10]. Briefly, the mice were cross-bred and are heterozygous for the human CETP transgene and for the LDL-receptor null allele (CETP^+/-^LDLr^-/+^). This transgenic mouse, as compared with the wild-type, exhibits a lipid profile closer to humans and hence, is more akin to a human model from a comparative physiological perspective. The mice were housed in a temperature and humidity controlled room (22 ± 1°C and 55-65%, respectively), with a 12 h light/dark cycle and free access to water and food (Nuvilab, Colombo, Paraná, Brazil).

Twenty-four 2-month-old male mice (19-20 g) were divided into four groups (n = 6/group): sedentary plus gum arabic (Sed-C), sedentary plus mesterolone (Sed-M), exercise plus gum arabic (Ex-C), and exercise plus mesterolone (Ex-M). The exercising mice (Ex-C and Ex-M) had one week of “preconditioning” (Monday through Friday) in which a low to moderate exercise level was performed consisting of treadmill running (0° inclination, 15 m/min, 20 min/day). Following the preconditioning period, the exercising mice were subjected to a graded high-intensity, aerobic running program, 5d/wk for 6 weeks (Table 1). The animals either received gum arabic (GA) or the dihydrotestosterone (DHT) derivative, mesterolone (M) (2 mg/kg body weight) (Proviron trademark of Schering, Schering do Brasil, São Paulo, SP, Brazil) by orogastric tube during the last 3 weeks of the study (3d/wk: Monday, Wednesday, and Friday). Mesterolone (1 alpha-methyl-17 beta-hydroxy-5 alpha-androstan-3-one) was chosen for two reasons: 1) it can be administered orally avoiding injection-induced wound formation and 2) as a non-17 alpha-alkylated derivative of testosterone it presents low hepatotoxicity. Gum arabic (compatible as a vehicle for the hydrophobic mesterolone) is inert, non-toxic, and has been used to improve absorption in the small intestine [11]. The amount of mesterolone administered per week (6 mg/kg) is considered to be a high dose (supra-therapeutic) [12] and is comparable to doses reportedly used in humans [13].

**Table 1:** Protocol for the treadmill running program (5d/wk, 6 wks) in CETP^+/-^LDLr^+/-^ transgenic mice, adapted from Smolka et al. [49].

Weeks	Velocity (m/min)	Duration (min)
1	12.42	20
2	14.70	30
3	16.68	45
4-6 ^(*)^	17.04	60

* Mesterolone (M) or gum arabic (vehicle) (2 µg/g body weight) was administered (Monday, Wednesday and Friday) by orogastric gavage during the last three weeks (4^th^ to 6^th^).

### Surgical procedures and body/muscle weights

Body weights were tracked weekly throughout the study (Table 2). At the end of the experimental period, fasted mice were anesthetized with a 1:1 mixture of ketamine chloride (Dopalen, 100 mg/kg of animal) and xylazine chloride (Anasedan, 10 mg/kg,) (2 μl/mg body mass, i.p.). Both anesthetics were purchased from Vetbrands (Jacareí, SP, Brazil). Soleus (SOL), tibialis anterior (TA), and gastrocnemius (GAS) muscles were carefully removed, weighed, and the animals were sacrificed with an overdose of the anesthetic. 

**Table 2:** Weekly body weight (BW) taken over the course of the study.

**BW(g**)	**1^week^**	**2^week^**	**3^week^**	**4^week^**	**5^week^**	**6^week^**	**7^week^**
**Groups**							
**Ex-M**	19.54±0.05	20.13±0.05	20.72±0.05	21.31±0.05	21.9±0.05	22.49±0.05	23.08±0.05 ^*^
**Ex-C**	19.54±0.05	20.04±0.04	20.54±0.05	21.04±0.05	21.54±0.05	22.04±0.06	22.54±0.06 ^* #^
**Sed-M**	19.54±0.04	20.18±0.04	20.83±0.04	21.47±0.03	22.12±0.05	22.77±0.04	23.41±0.04
**Sed-C**	19.54±0.02	20.18±0.02	20.82±0.03	21.46±0.04	22.1±0.04	22.74±0.04	23.38±0.02 ^#^

The same symbol in each column indicates a significant difference between groups (P≤0.05). Values given are mean ± SD.

### Muscle fiber typing

**Figure 1:**
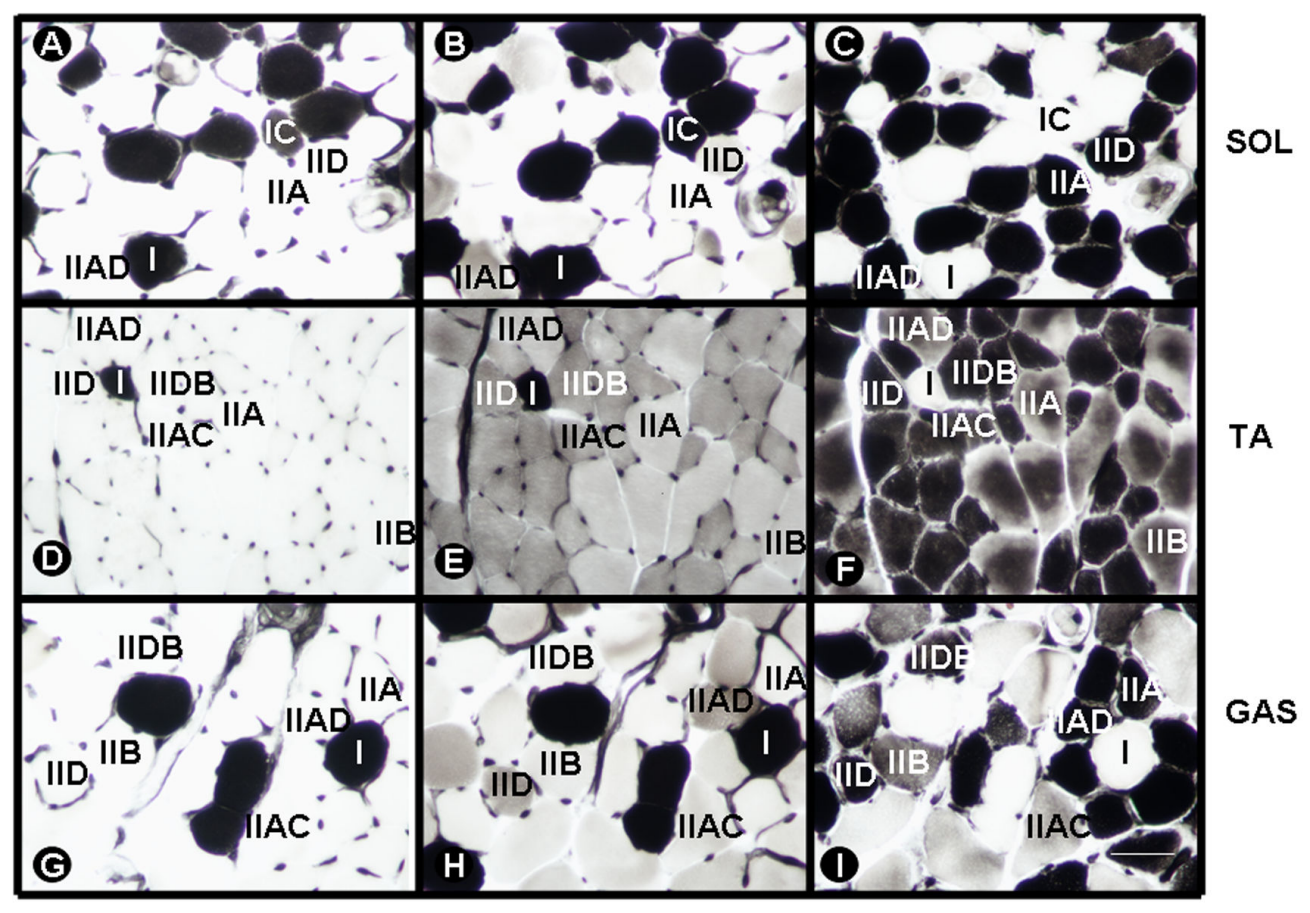
mATPase histochemical muscles preparations. Serial cross sections from soleus (SOL) (A,B,C), tibialis anterior (TA) (D,E,F), and gastrocnemius (GAS) (G,H,I) muscles demonstrating fiber type delineation using mATPase histochemistry after preincubation at pH 4.3 (A, D, G), 4.5 (B, E, H), and 10.5 (C, F, I). I, type I; IC, type IC; IIAC, type IIAC; IIA, type IIA; IIAD, type IIAD; IID, type IID; IIDB, type IIDB; IIB, type IIB. Bar = 50 µm.

The middle portion of each muscle was separated, oriented in a mixture of gum tragacanth (Sigma, St. Louis, MO, USA) and Tissue-Tek embedding medium (Sakura Finetechnical Co., Tokyo, Japan), immediately frozen in isopentane cooled to -156°C in liquid nitrogen, and stored at -70°C. Transverse cross sections (12 µm thick) were obtained in a cryostat, collected on coverslips, and stored frozen at -40°C until all samples were ready for processing. Fiber types were identified using myofibrillar adenosine triphosphatase (mATPase) histochemistry [14] following pre-incubation at pH 4.3, 4.5 [15] and 10.5 [16] (Figure 1). In some cases, the alkaline pH was slightly adjusted to optimize the reaction (10.57 and 10.6 for TA and GAS muscles, respectively). The muscle sections obtained following pre-incubation at pH 4.5 were photographed and mounted as a plate. This plate was then compared with the other slides to identify the entire range of fiber types (I, IC, IIAC, IIA, IIAD, IID, IIDB, and IIB) [17] (Figure 1). 

### Histomorphometry

Image-Pro Express software (Media Cybernetics, Silver Spring, MD, USA) was used to calculate cross-sectional areas (CSA) using a BX 51 Q Color 3 Olympus light microscope (Olympus, Japan). A minimum of 80 muscle fibers per type/per muscle was analyzed in each group. The fields were randomly selected, and all muscle fibers encompassed in these fields were evaluated. Insufficient numbers of hybrid fiber types IC and IIAC were found to obtain statistically meaningful cross-sectional values.

### Statistical analysis

All numerical data are presented as mean ± standard deviation (SD). A one-way analysis of variance (ANOVA) followed by Tukey’s post hoc test was used to compare differences between groups. If an overall ANOVA revealed significant differences, a two-way ANOVA was used to determine how mesterolone treatment or exercise influenced body/muscle weight, fiber type composition or CSA changes, and if there was an interaction between training vs. mesterolone. Differences were considered significant at P<0.05. Origin software package (Microcal^TM^ Software Inc., Northampton, MA, USA) was used for all statistical analyses. 

## Results

### The effect of mesterolone treatment on body weight (BW)

There was a tendency for BW to gradually increase throughout the trial period for all groups (Table 2). However, the smallest overall weight gain was achieved by the training mice treated only with gum arabic (Ex-C). Mesterolone treatment appeared to counteract the attenuated weight gain effect of exercise. As a result, the body weight gain of Ex-M was similar to both sedentary groups (Sed-C and Sed-M). At the end of the sixth week, Ex-C and Ex-M differed significantly from the Sed-C and Sed-M, respectively in relation to the final body weight (P<0.05) (Table 2). Two-way ANOVA showed that there was no interaction between mesterolone and exercise for BW values.

### The effect of mesterolone treatment on muscle wet weight (MW)

The wet weights of all three muscles (soleus, tibialis anterior, and gastrocnemius) were smallest in sedentary animals treated with gum arabic (Sed-C) and largest in exercised animals treated with mesterolone (Ex-M) (Table 3). The effect of mesterolone in sedentary mice (Sed-C vs. Sed-M) was stronger than in exercised mice (Ex-C vs. Ex-M) suggesting exercise blunted the steroid effect. The greatest impact of mesterolone on muscle weight, both in sedentary and exercised mice, was in TA (39% and 20% increase, respectively) and the smallest impact in GAS (16% and 12%, respectively) (Table 3). The effect of exercise per se in promoting a significant increase in MW was comparable among the muscles and was stronger than induced by mesterolone (with the exception of TA: compare Ex-C vs. Sed-C and Sed-M vs. Sed-C). Two-way ANOVA showed that there was no interaction between mesterolone and exercise for MW. Exercise plus mesterolone appeared to have an additive effect, such that the largest muscle wet weights were obtained by the Ex-M group for all three muscles.

**Table 3:** Muscle wet weights (g) of soleus (SOL), tibialis anterior (TA) and gastrocnemius (GAS) muscles from transgenic mice.

	SOL	TA	GAS
Ex-M	0.020 ± 0.002 ^† ‡^	0.067 ± 0.002 ^† ‡^	0.077 ± 0.001 ^† ‡^
Ex-C	0.017 ± 0.001 ^# †^	0.056 ± 0.002 ^# †^	0.069 ± 0.001 ^# †^
Sed-M	0.015 ± 0.001^* ‡^	0.057 ± 0.006 ^* ‡^	0.057 ± 0.004^* ‡^
Sed-C	0.012 ± 0.004^* #^	0.041 ± 0.001^* #^	0.049 ± 0.001^* #^

The same symbol in each column indicates a significant difference between groups (*P*<0.05). Values given are mean ± SD.

### The effect of mesterolone treatment on muscle fiber type distribution

SOL muscles from all groups of mice contained a predominance of type I and IIA fibers, with type IID representing the minority (Table 4). On the other hand, the two fast muscles (TA and GAS) contained predominantly fiber types IID, IIDB, and IIB, with the more oxidative fibers (types I, IIA, and IIAD) found in low numbers (Table 4). Type IC or IIAC fibers were rare in these fast muscles. In the SOL, mesterolone treatment caused significant increases in the hybrid fiber populations (IC, IIAC, and IIAD) and type IID, with a concomitant decrease in the type IIA (Table 4). This effect was blunted by exercise. On the other hand, the various treatments (exercise, mesterolone, and the combination of both) had a minimal impact on fiber type distribution in the predominantly fast muscles (TA and GAS) (Table 4). In the TA, the high-intensity aerobic exercise per se did cause small but significant increases in the proportion of fiber types IIA and IIAD (Sed-C vs. Ex-C). Similarly in the TA, mesterolone treatment combined with exercise resulted in small but significant increases in the percentage of fiber types I, IIA and IIAD compared to mesterolone treatment alone (Ex-M vs. Sed-M) (Table 4). In GAS, the aerobic exercise program resulted in a significant increase in the percentage of fiber type IID, with concomitant decreases in IIAD and IIDB (Table 4). Fiber type changes in GAS were also found comparing Sed-M vs. Ex-M groups: there was decrease in the IIAD and IIDB, with concomitant increases in the proportion of IIB (Table 4). Two-way ANOVA showed that there was an interaction between mesterolone and exercise for fiber type IC (P=0.0339); IIAC (P=0.0291); IIA (P =0.0258); IIAD (P=0.0424) and IID (P=0.0190) for SOL, and for fiber type IIDB (P =0.0164) and IIB (P=0.0391) for GAS.

**Table 4:** Muscle fiber type percentages of *soleus* (SOL), tibialis anterior (TA) and *gastrocnemius* (GAS) muscles from transgenic mice.

**SOL**	**I**	**IC**		**IIAC**		**IIA**		**IIAD**		**IID**		
**Group**												
**Ex-M**	41.7± 5.2	12.6±5.3		10.1±3.5		27.6±2.7		5.9±2.4		2.1±0.7		
**Ex-C**	38.0± 5.4	8.7± 1.5		8.6±3.1		35.8±8.4		5.3±3.1		3.7±2.4*		
**Sed-M**	37.5± 2.4	13.8±3.0*		14.3±5.1*		23.3± 6.6*		9.6±2.3*		1.6±0.6**^#^**		
**Sed-C**	39.9±2.2	3.1±3.7*		4.9±4.6*		47.6±12.2*		4.0±3.3*		0.5±0.2*^#^		
**TA**	**I**	**IC**		**IIAC**		**IIA**		**IIAD**		**IID**	**IIDB**	**IIB**
**Group**												
**Ex-M**	1.1±0.2*	**-----**		**-----**		5.8±1.8*		8.7±1.8*		37.5±4.0	24.4±5.0	22.5±4.4
**Ex-C**	0.8 ±0.2	**------**		**------**		5.1±1.0 ^#^		8.4±1.1 ^#^		43.3±5.8	20.8±4.7	21.6±6.6
**Sed-M**	0.7±0.2*	**-----**		**-----**		3.6±0.3*		5.8±1.3*		43.0±5.7	27.1±4.4	19.8±6.9
**Sed-C**	0.6±0.2	**-----**		**-----**		3.1±0.2 ^#^		4.7±0.4 ^#^		46.4±3.6	25.4±4.6	19.9±3.8
**Gas**	**I**	**IC**		**IIAC**		**IIA**		**IIAD**		**IID**	**IIDB**	**IIB**
**Group**												
**Ex-M**	3.4±1.5	**-----**		**-----**		2.3±0.8		3.2±1.7*		25.5±5.3	6.6±2.2**^#^**	59.1±4.0 *
**Ex-C**	3.1±1.6	**-----**		**-----**		2.4±1.0		1.7±0.7 ^#^		33.0±5.4*	8.7±2.6*	51.2±7.9
**Sed-M**	4.1±1.4	**-----**		**-----**		3.5±0.9		7.1±2.2*		19.7±3.9	23.1±4.1**^#^**	42.6±9.1*
**Sed-C**	4.2±1.9	**-----**		**-----**		2.2±1.4		5.2±1.7 ^#^		23.0± 3.9 *	18.2±3.9*	47.1±5.6

### The effect of mesterolone treatment on muscle fiber cross-sectional area (CSA)

SOL - In the SOL muscle, the oxidative fibers (I and IIA) had the largest mean fiber area for all groups (Table 5). Compared to the other groups, SOL of Sed-C showed the least variability in fiber type cross-sectional areas among the different fiber types. The administration of mesterolone caused significant increases in the CSA of fiber types I, IIA, and IID in the sedentary mice (comparing Sed-C vs. Sed-M) (Table 5). The high-intensity exercise per se or in steroid-treated mice (Sed-C vs. Ex-C or Sed-M vs. Ex-M) also caused large, significant increases in the sizes of all major fiber types in SOL. With the exception of slightly larger type I fibers (7%), the cross-sectional areas of fiber types IIA, IIAD, and IID were similar between Ex-C and Ex-M. In the SOL, type I fibers appeared to be the most responsive to the various treatments, whereas type IIAD was the least. Two-way ANOVA showed that there was an interaction between mesterolone and exercise only for type I (P=0.0028).

**Table 5:** Cross-sectional area (µm^2^) of the major fiber types from *soleus* (SOL), tibialis anterior (TA) and *gastrocnemius* (GAS) muscles.

**SOL**	**I**	**IIA**	**IIAD**	**IID**		
**Group**						
**Ex-M**	1988± 14.0**^#†^**	1806.6±17.2^*^	1284±17.7**^#^**	1357.2±37.3**^#^**		
**Ex-C**	1858.6± 9.3^*†^	1744.7±36.6**^&^**	1168.7±42.1^*^	1308.5±25.8^*^		
**Sed-M**	1234.3±27.3**^#&^**	1124.6± 4.4^*†^	942.5±13.2**^#^**	1029.9±15.1**^#†^**		
**Sed-C**	923.3±13.4^*&^	948.9±11.7**^†&^**	850.51±22.2^*^	839.5±5.8^*†^		
**TA**	**I**	**IIA**	**IIAD**	**IID**	**IIDB**	**IIB**
**Group**						
**Ex-M**	848.2±13.7*	895±24.9*	933.8±18.3* **^†^**	924.8±16.5**^†^**	1114±33.1*^&^	2555.7±78.0*
**Ex-C**	706.1±65.0**^#^**	827.9±25.8 ^#^	765.3±19.1 ^#†^	836.6±16.5*	928.1±22.5**^&^**	2301.2±19.8**^#^**
**Sed-M**	456.9±1.9*^†^	553.8±14.1*	783.4±7.8 *^&^	687.3±16.6**^†&^**	811.7±15.1*	2093.5±29.3*
**Sed-C**	400.7±2.6**^#†^**	508.9±19.2**^#^**	474.6±6.1 ^#**&**^	529.3±26.6*^&^	818±21.0	1943±44.5**^#^**
**GAS**	**I**	**IIA**	**IIAD**	**IID**	**IIDB**	**IIB**
**Group**						
**Ex-M**	971.5±31.6**^#&^**	752.5±5.5**^#†^**	934.2±19.8 * **^†^**	1117.8±45.4**^†#^**	949.3±39.8	2346.4±16.4*^†^
**Ex-C**	810.5±33.0*^&^	649.2±18.3**^†&^**	728±5.0 ^# **†**^	779.2±8.8*^#^	830.5±22.0	2157.2±25.3**^†&^**
**Sed-M**	662.4±3.3**^#†^**	574.2±10.7*^#^	680.8±4.7 * **^&^**	830.5±21.0**^†&^**	821.9±18.4	2024.1±13.5*^#^
**Sed-C**	490±3.0*^†^	480±3.0*^&^	576±13.5 ^#**&**^	654.8±6.5*^&^	767.9±6.1	1798.7±20.4**^#&^**

The same symbol in each column indicates a significant difference between groups (*P*<0.05). Data are presented as mean ± SD.

TA - In TA of Sed-C, there was a hierarchy of fiber size from largest to smallest such that: IIB>IIDB>IID=IIA=IIAD>I (Table 5). Compared to Sed-C, mesterolone administration in sedentary mice (Sed-M) caused significant hypertrophy of fiber types I, IIAD, and IID. In contrast, AAS treatment in trained mice (Ex-C vs. Ex-M) caused significant increases in the cross-sectional areas of only types IIAD and IIDB. For the most part, exercise per se (Sed-C vs. Ex-C) or in mesterolone-treated mice (Sed-M vs. Ex-M) appeared to have a similar effect on the cross-sectional areas of the different fiber types in TA causing increases in the sizes of fiber types I, IIA, IIAD, IID, IIDB and IIB (Table 5). A two-way ANOVA showed there was an interaction between mesterolone and exercise for fiber types IIAD (P=0.0041) and IIDB (P =0.0144).

GAS – GAS of Sed-C displayed a hierarchy of fiber size from largest to smallest similar to TA: IIB>IIDB>IID>IIAD>I=IIA (Table 5). When compared to Sed-C, the GAS of both Sed-M and Ex-C had significantly larger areas for all fiber types (I, IIA, IIAD, IID, and IIB) except IIDB (Table 5). The combined effects of exercise + mesterolone treatment (Ex-M) caused additional hypertrophy such that when comparing Ex-C vs. Ex-M or Sed-M vs. Ex-M significantly larger areas were found for these same fiber types (I, IIA, IIAD, IID, and IIB). In general, the most responsive fibers to all the stimuli in GAS were type I and IID (Table 5). A two-way ANOVA showed that there was an interaction between mesterolone and exercise for only fiber type IIAD (P =0.0158).

## Discussion

Professional and recreational athletes continue to use AAS with the hope of enhancing performance by increasing muscle mass and strength [4]. Interestingly, there exists relatively little research concerning the muscular effects of AAS administration, and to the authors’ knowledge, no data are available regarding the effects of AAS on fiber type composition in mice. Continued research in this area could yield important information regarding the impact of AAS on skeletal muscle fiber type distribution and size, giving insight into potential AAS-induced alterations in phenotypic profile. For this purpose, the present study meticulously delineated the entire range of mATPase-based fiber types utilizing a transgenic mice specifically engineered to express a lipid profile similar to humans. . The present study used a 6-week, high-intensity treadmill running program (typically catabolic in nature) to evaluate the interaction of a 3-week anabolic-androgenic oral program known to promote body and muscle mass gains. 

### Body and muscle mass

In this study, all animals gained body weight (BW) over the course of the 6-week program. However, the BW gain of the exercising mice (both with and without AAS supplementation) was minimized compared to the control groups. These data are consistent with studies demonstrating aerobic exercise plays a key role in body weight control [4]. In addition, the anabolic effect of mesterolone in exercised mice appeared to play a role in counteracting this catabolic effect. 

The high-intensity, aerobic exercise caused a significant increase in muscle wet weights with an additional increase following mesterolone treatment. In all three muscles, the effect of mesterolone in sedentary mice was stronger than in exercised mice suggesting that the effect of exercise partially blunted the anabolic effect of mesterolone. Skeletal muscles express a variety of different types of proteins associated with metabolism, inflammation, and contractile activity in response to exercise. A large number of different systemic and muscular proteins could also result from anabolic-androgenic steroid treatment. 

Although the elucidation of the complex molecular mechanisms underlying the ergogenic effects of AAS treatment was not the aim of the present study, muscle function may be improved by increasing protein synthesis or membrane stabilization [18]. This may be accomplished via a competitive occupancy of glucocorticoid receptors by AAS which would act antagonistically to the catabolic action of glucocorticoid hormones. In the present study, adaptive differences observed among the three muscles examined implies alternative binding of the steroid to a number of androgen receptors resulting in agonistic promotion of skeletal muscle protein synthesis [19,20]. Androgens receptors (AR) are expressed in satellite cells, differentiated myofibers, intramuscular fibroblasts, and different types of motoneurons. In addition, the regulation of plasmatic levels of insulin-like growth factor-1 (IGF-1), growth hormone, and thyroid hormone (as well as, the antagonism of glucocorticoids) are roles linked to anabolic-androgenic signaling [see 21]. 

The effects of the combination of mesterolone treatment and exercise on skeletal muscle of CETP transgenic mice are unknown. Previous studies utilizing CETP mice have shown that the plasmatic level of thyroid hormones influences lipoprotein metabolism. High levels of thyroid hormone increase CETP activity and hence, cause an increase in the plasmatic levels of HDL-cholesterol [22]. In addition, it has been shown that patients with hyperthyroidism tend to show low levels of LDL-c, VLDL-c and HDL-c [23]. A previous study from our laboratory using this same transgenic mouse model has shown that mesterolone treatment in sedentary mice caused an increase in total cholesterol, triglycerides, LDL-c and VLDL-c, whereas the combination of AAS treatment and exercise resulted in the reverse (i.e., an increase in HDL-c and decreases in LDL-c, VLDL-c and triglycerides) [24]. Taken together, these data suggest the effect of AAS treatment on muscle growth and the additive hypertrophic effect following exercise may be the result of a transcriptional program involving thyroid regulation, IGF-1 and its splice variant mechano growth factor (MGF) [21]. Such a transcription program would likely differ on the basis of the metabolic and twitch characteristics of the muscle. 

### Fiber type distribution

To date, studies investigating the effects of AAS on muscle fiber type composition have reported equivocal results. Although comparisons between the various studies are difficult, most research in this area has shown no effect of AAS administration on fiber type distribution or relative myosin heavy chain isoform content in various muscles from both human [25-28] and rat [12,23-32]. A few studies have, however, documented AAS-induced alterations in fiber type composition. 

Holmäng et al. reported a decrease in the relative number of type I fibers with a concomitant increase in type II in the soleus muscle and an increase in the proportion of type II fibers in the deep, red portion of the gastrocnemius following testosterone treatment in oophorectomized female rats [33]. Likewise, Salmons reported a decrease in the population of “intermediate” fibers with an increase in the proportion of “white” (glycolytic) fibers in female rabbit tibialis anterior muscle treated with AAS [34]. This apparent AAS-induced transformation in the slow-to-fast (oxidative→glycolytic) direction appears to be at odds with studies reporting fiber type adaptations in the opposite direction. Egginton and Dimauro et al. found an increase in the proportion of FOG (fast oxidative-glycolytic) fibers with a concomitant decrease in FG (fast glycolytic) fibers in various muscles from female rats following chronic administration of AAS [35,36]. Indeed, this increase in the number of oxidative fibers may explain the significant improvement in EDL fatigue resistance after 5-6 weeks of AAS treatment [35]. These data have more recently been supported by Fontana et al. [37]. Fontana et al. reported a shift into an even slower profile in soleus m. from mice following AAS treatment: significant increase in the number of capillaries and mitochondria per muscle fiber and increased proportion of type I (slow) fibers and a concomitant decrease in the proportion of type II (fast) [37]. The present study supports and extends these findings with conversions in the fast-to-slow direction found in the soleus muscle after AAS administration and high-intensity exercise. 

### Hypertrophy

Variable results have also been documented regarding the impact of AAS administration on muscle and fiber size. Although some studies have reported no change [29,36,38,39] or even a decrease [14] in fiber size following steroid treatment, most have demonstrated a significant increase in muscle mass and fiber size [12,25,28,34,37,40,41]. The present study supports an AAS-induced hypertrophic effect in all three muscles, regardless of their metabolic/contractile profile. 

AAS administration has been shown to increase muscle protein synthesis [42,43] and ultimately muscle mass. This increase in muscle mass is predominantly the result of muscle fiber hypertrophy [28] and involves satellite cell activation and incorporation into the muscle fiber [44]. In the present study, mesterolone treatment alone resulted in hypertrophy of most fiber types and significant increases in the wet weights of all three muscles. Compared to AAS-treatment alone, the high-intensity exercise protocol caused comparable increases in fiber sizes and wet weight of all three muscles. However, the combination of high-intensity, endurance exercise and mesterolone treatment resulted in an additive hypertrophic effect in fiber sizes and muscle wet weights. This enhanced hypertrophy induced by steroid use in conjunction with exercise has been previously reported in strength-trained athletes [25,27,45-47] and in high-intensity trained rats [36,37]. No doubt the dramatic increase in muscle mass is a contributing factor to improved performance [13,46]. 

### Perspectives

Overall, it is difficult to explain such varied findings concerning the effects of AAS treatment on fiber type distribution and size. Conflicting evidence may be due to a variety of factors: species, muscles chosen, androgen receptor density, fiber typing methodology, dosage/type of AAS, mode of administration, study duration, gender, age, and activity level [18,26,48]. In the present study, we were able to detect subtle changes in fiber type distribution with the utilization of refined mATPase histochemistry [17] by delineating the entire range of pure/hybrid fiber types. Previous AAS studies have separated fibers into either two [27-29,33,37] or three [12,35,36] broad categories, and were thus, likely unable to detect subtle changes in fiber type composition. Data from the present study suggest a differential AAS response between slow and fast muscles. Mesterolone induced differential adaptive changes in the transgenic mice hindlimb muscles amounting to significant fast-to-slow fiber type transformations in the slow soleus muscle with minimal effect on the predominantly fast muscles. Mesterolone and exercise each induced comparable increases in the size of all major fiber types in all three muscles. However, AAS plus exercise caused a cumulative effect resulting in additional hypertrophy. These data were supported by similar increases in the muscle wet weights. The findings show that in this transgenic murine model the caloric expenditure induced by a metabolically-demanding exercise program was superimposed by protein synthesis resulting in muscle mass gains, which were potentiated in trained animals treated with mesterolone. Data obtained from this transgenic model (specifically engineered to express a lipemic phenotype akin to humans) could be relevant to humans from a comparative perspective. 

## Figures and Tables

**Table:** 

	
